# Muscle atrophy induced by SOD1^G93A ^expression does not involve the activation of caspase in the absence of denervation

**DOI:** 10.1186/2044-5040-1-3

**Published:** 2011-01-24

**Authors:** Gabriella Dobrowolny, Michela Aucello, Antonio Musarò

**Affiliations:** 1 Institute Pasteur Cenci-Bolognetti, DAHFMO-Unit of Histology and Medical Embryology, IIM; Sapienza University of Rome, Via A. Scarpa, 14 Rome I-00161, Italy; 2 School of Biomedical & Sports Science; Unit of Human Biology, Edith Cowan University, Western Australia, Australia

## Abstract

**Background:**

The most remarkable feature of skeletal muscle is the capacity to adapt its morphological, biochemical and molecular properties in response to several factors. Nonetheless, under pathological conditions, skeletal muscle loses its adaptability, leading to atrophy or wasting. Several signals might function as physiopathological triggers of muscle atrophy. However, the specific mechanisms underlying the atrophic phenotype under different pathological conditions remain to be fully elucidated. In this paper, we address the involvement of caspases in the induction of muscle atrophy in experimental models of amyotrophic lateral sclerosis (ALS) expressing the mutant SOD1^G93A ^transgene either locally or ubiquitously.

**Results:**

We demonstrate that SOD1^G93A^-mediated muscle atrophy is independent from caspase activity. In particular, the expression of SOD1^G93A ^promotes a reduction of the phosphatidylinositol 3-kinase/Akt pathway associated with activation of forkhead box O3. In contrast, the activation of caspases occurs later and is causally linked to motor neuron degeneration, which is associated with exacerbation of the atrophic phenotype and a shift in fiber-type composition.

**Conclusion:**

This study suggests that muscle atrophy induced by the toxic effect of SOD1^G93A ^is independent from the activation of apoptotic markers and that caspase-mediated apoptosis is a process activated upon muscle denervation.

## Introduction

The decline in functional performance and restriction of adaptability represents the hallmark of skeletal muscle and brain pathologies. A crucial system severely affected in several neuromuscular diseases is the loss of effective connection between muscle and nerve, leading to a pathological noncommunication between the two tissues.

Among degenerative diseases, amyotrophic lateral sclerosis (ALS) represents one of the best examples in which the interplay between muscle and nerve is severely compromised [1,2]. The most typical feature of this progressive lethal disease is the degeneration of motoneurons, muscle weakness, fasciculations, muscle atrophy, speech and swallowing disabilities, progressive paralysis and death caused by respiratory failure. Although a significant proportion of familial ALS results from a toxic gain of function associated with dominant superoxide dismutase 1 (SOD1) mutations, the etiology of the disease and its specific cellular origins have remained difficult to define.

The best-known function of SOD1 is to detoxify cells from accumulation of free radicals, which represent a toxic by-product of mitochondrial oxidative phosphorylation [3]. The obvious loss of motor neurons in the spinal cord initially focused attention on how mutant SOD1 may act within motor neurons to provoke neuronal degeneration and death. However, the mutant gene products are expressed widely, raising the possibility that the toxic cascade may be achieved wholly or in part by mutant SOD1 action in non-neuronal cells. This notion is supported by recent works. Notably, transgenic mice in which mutant SOD1 was largely restricted to neurons developed disease only at an old age [4]. However, the disease progressed slowly without reaching the same degree of paralysis as the classical animal model of ALS, in which the same mutant SOD1 gene is ubiquitously expressed [5]. Although mutant SOD1 is also expressed by muscle, it is not clear whether its presence in skeletal muscle directly contributes to any pathological sign of ALS.

This issue has been investigated recently by our group, demonstrating that muscle selective expression of SOD1 mutation was sufficient to induce muscle atrophy associated with significant reduction in muscle strength, sarcomere disorganization and significant changes in mitochondrial morphology [6]. These data, along with other evidence [7-10], might explain how the ubiquitous expression of SOD1^G93A ^first causes muscle atrophy, which is later followed by alteration of the neuromuscular junction (NMJ), retrograde axonal degeneration and last motor neuron death. This retrograde and progressive (muscle to NMJ to nerve) sequential pattern of degeneration suggests the possibility that certain muscle abnormalities indeed precede motor neuron death rather than result from it.

The characteristic loss in muscle mass, coupled with a decrease in strength and force output, modulation in brain metabolism and alteration in oxidative stress, has been associated with a selective activation of apoptotic pathways and a general reduction in survival mechanisms. Indeed, several studies have indicated the activation of apoptotic events in pathological conditions associated with muscle atrophy, including neuromuscular diseases and muscle disuse [11]. However, whether apoptosis is a trigger or a result of muscle atrophy in ALS is an unanswered question.

The central component and the executor of the apoptotic machinery in several tissues is a proteolytic system involving a family of cysteine proteases called caspases [12]. Caspases are all expressed as inactive proenzymes with a molecular weight between 50 and 33 kDa and are activated after cleavage at the aspartate residues, generating the active product with lower molecular weight.

In this paper, we extend a previous study characterizing the involvement of caspases in the induction of muscle atrophy exerted by local and ubiquitous expression of the SOD1^G93A ^transgene. We reported that muscle atrophy in myosin light chain (MLC)/SOD1^G93A ^mice does not involve the activation of caspase-1 and caspase-3, which represent the initiator and final effector of cell death, respectively. Of note, the expression and activity of caspase-1 and caspase-3 increased in the muscle of the classical animal models of ALS [5], which express the SOD1^G93A ^gene ubiquitously, but only at the paralysis stage.

In contrast, muscle atrophy in MLC/SOD1^G93A ^mice is the result of deregulation of the Akt pathway, which leads to a reduction in the activity of molecular markers of protein synthesis, such as mammalian target of rapamycin (mTOR) and p70s6K, and to the activation of proteolytic mechanisms mediated by forkhead box O3 (FoxO3) activity.

## Results

### Caspases are activated in muscle upon motor neuron degeneration

We have determined the potential activation of apoptotic pathways in MLC/SOD1^G93A ^transgenic mice. Critical players in apoptosis-mediated muscle atrophy are caspases (cysteine-dependent, aspartate-directed proteases). In particular, caspase-3 is a critical executioner of apoptosis; it is translated as an inactive 37-kDa proenzyme that is activated after cleavage, generating a low-molecular-weight active product (p17/p19) [13].

**Figure 1:**
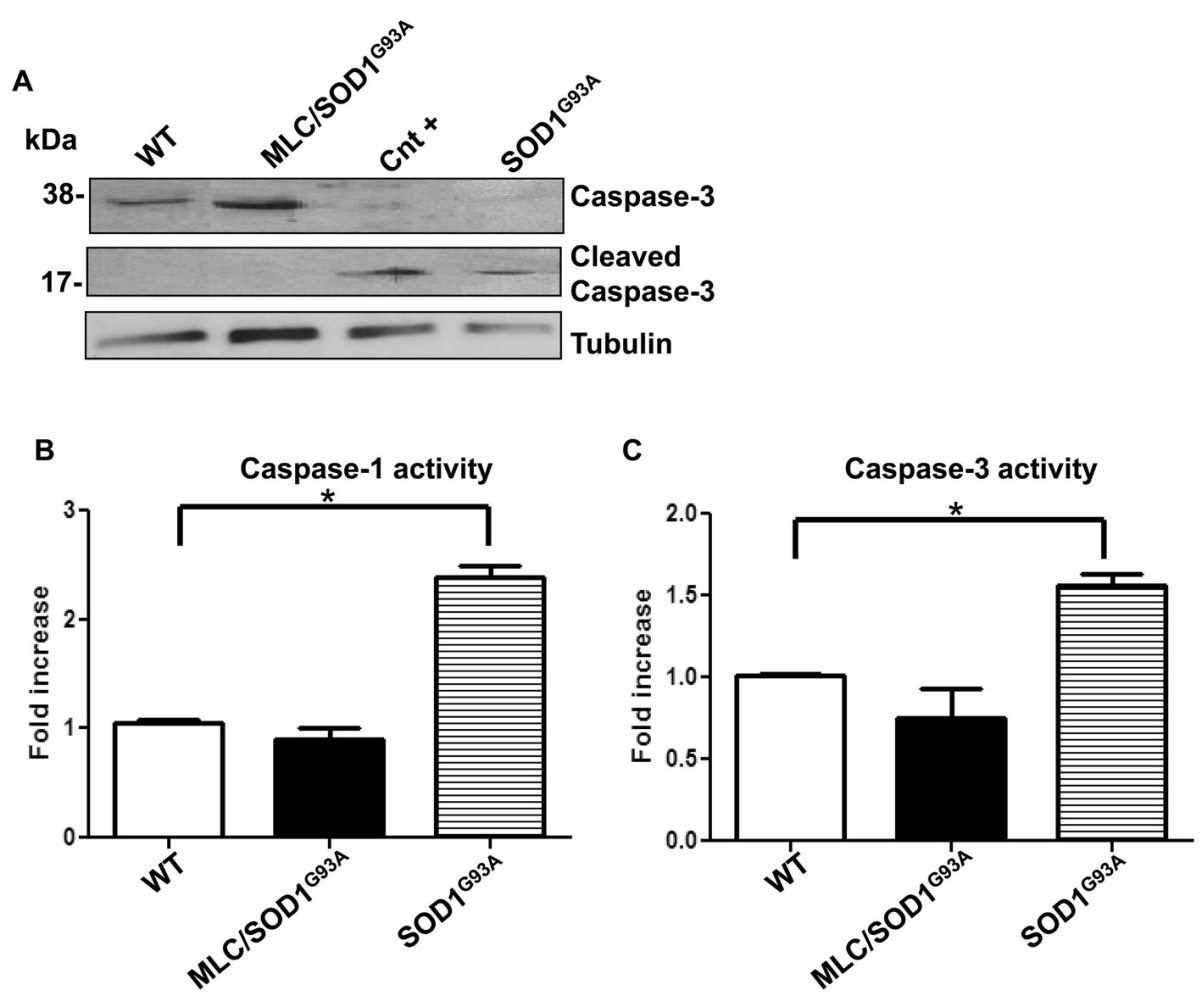
**Caspase expression and activity are selectively upregulated in atrophic muscle of SOD1^G93A ^mice at paralysis stage**. (**A**) Representative Western blot analysis using caspase-3 antibody (top) and cleaved caspase-3 antibody (bottom) in wild-type (WT) (lane 1), myosin light chain (MLC)/SOD1^G93A ^(lane 2) and SOD1^G93A ^(lane 4) transgenic muscle. Cnt^+ ^represents the positive control (HeLa cells treated with staurosporin) for cleaved caspase-3. Immunoblotting for α-tubulin served as a control for protein loading. (**B and C**) Analysis (n = 5) of (**B**) caspase-1 and (**C**)caspase-3 activity on WT, MLC/SOD1^G93A ^and SOD1^G93A ^transgenic mice. *P < 0.05. Mean values ± SEM for caspase-1: WT = 1.055 ± 0.03, MLC/SOD1^G93A ^= 0.908 ± 0.10 and SOD1^G93A ^= 2.385 ± 0.10. Mean values ± SEM for caspase-3: WT = 1.01 ± 0.01, MLC/SOD1^G93A ^= 0.75 ± 0.18 and SOD1^G93A ^= 1.56 ± 0.07.

Western blot analysis for the total and the cleaved form of caspase-3 expression (Figure 1A) performed on 16-week-old wild-type and MLC/SOD1^G93A ^skeletal muscle did not reveal a significant difference between the two experimental models, suggesting that at this stage, apoptosis does not play a critical and pivotal role in the induction of muscle atrophy mediated by reactive oxygen species accumulation. It has been reported that expression of apoptosis-related proteins increases in denervated muscle atrophy [10,14-16], raising the prospect that apoptosis is not a mechanism necessarily involved in the induction of muscle atrophy, but rather might be a later event associated with denervation.

To support this hypothesis, we analyzed and compared caspase expression and activity in the muscles of both MLC/SOD1^G93A ^[6] and SOD1^G93A ^mice [5] at 123 days of age. Of note, the SOD1^G93A ^mouse represents the classical animal model of ALS; it does not display any pathological sign of the disease until age 111 ± 1.8 days, which is considered the disease onset [5,17], whereas motor neuronal degeneration and complete muscle paralysis occurs at age 123 days [5,17]. As shown in Figure 1A, the cleaved active product of caspase-3 selectively increased in the paralyzed SOD1^G93A ^muscle.

To further verify the involvement of caspases on muscle wasting associated with motor neuron degeneration, we evaluated the activity of caspase-1 and caspase-3 in wild-type, MLC/SOD1^G93A ^and SOD1^G93A ^muscles. Figures 1B and 1C show that the activity of caspase-1 and caspase-3 increased in SOD1^G93A ^mice at 123 days of age, which corresponds to the stage of motor neuron degeneration and paralysis. In contrast, in younger presymptomatic SOD1^G93A ^mice (age 90 days), caspase-1 and caspase-3 activity did not show significant differences compared to wild-type and MLC/SOD1^G93A ^mice (data not shown). These data suggest that muscle atrophy associated with either local or ubiquitous expression of the toxic SOD1^G93A ^protein is independent of caspase activation, whereas caspase-mediated apoptosis was greater in paralyzed muscle.

### Muscle atrophy is independent from motor neuron degeneration

The evidence that caspase activity increases upon muscle denervation and that local expression of SOD1^G93A ^did not induce significant changes in the number of motor neurons [6], suggests that muscle atrophy associated with muscle expression of SOD1^G93A ^is causally linked to the direct toxic effect of mutant SOD1 protein rather than to muscle denervation.

To support this evidence, we analyzed relevant markers of nerve activity. In particular, nerve activity has a major role in the maintenance and modulation of fiber-type properties by selective activation of fiber-specific gene expression [18]. A shift in fiber composition therefore constitutes a marker associated with muscle denervation [18]. Specifically, motor neuron degeneration and muscle denervation cause a shift from slow to fast muscle phenotype.

**Figure 2:**
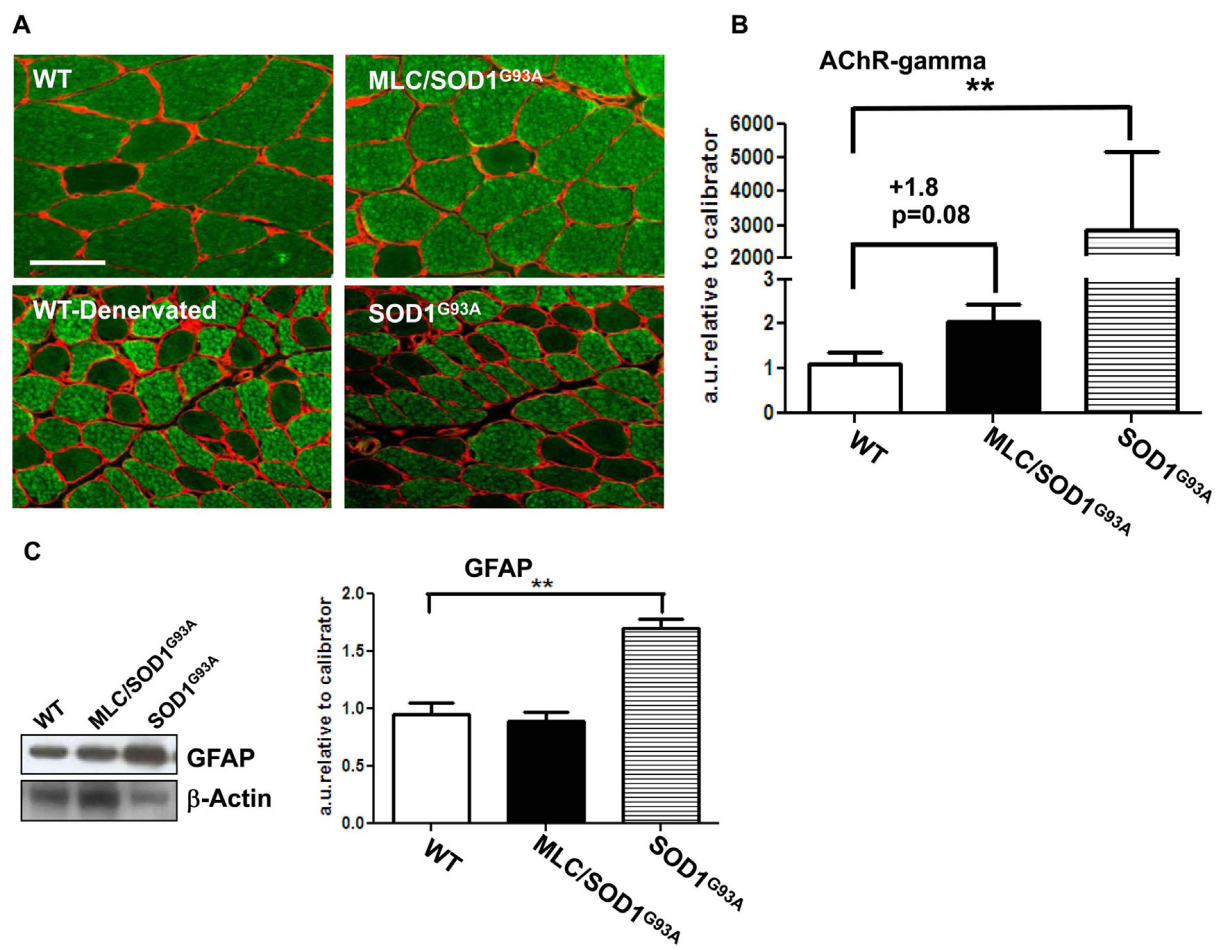
**Muscle atrophy is exacerbated in denervated mice**. (**A**) Immunofluorescence analysis of myosin heavy chain (MyHC)-slow (green) and laminin (red) performed on soleus muscles of WT, MLC/SOD1^G93A^, WT-denervated and SOD1^G93A ^muscle at age 123 days, which corresponded to the paralysis stage in SOD1^G93A ^mice. Bar, 50 μm. (**B**) Real-time polymerase chain reaction (PCR) for nicotinic acetylcholine receptor (AChR)-γ expression in both WT and MLC/SOD1^G93A ^transgenic muscle. **P = 0.006. (**C**) Representative Western blot analysis (left) for glial fibrillary acidic protein (GFAP) expression in the spinal cord of WT, MLC/SOD1^G93A ^and SOD1^G93A ^transgenic mice at 123 days old. Immunoblotting for β-actin served as a control for protein loading. Right: Densitometric analysis of seven separate immunoblot experiments showing the expression levels of GFAP calculated as ratios of GFAP/β-actin expression. **P < 0.005.

Immunohistochemical analysis (Figure 2A) for the expression of myosin heavy chain-slow (MyHC-slow) revealed that fiber-type composition was unchanged in the soleus muscle of both wild-type and MLC/SOD1^G93A ^mice. In contrast, fiber-type composition was altered in both denervated wild-type soleus muscle and SOD1^G93A ^soleus muscles at paralysis stage, with a shift toward a fast fiber type (Figure 2A).

Notably, the denervated muscles of wild-type mice and the paralyzed muscle of SOD1^G93A ^mice displayed a more severe atrophic phenotype, as indicated by the reduction in fiber diameter (Figure 2A). This suggests that motor neuron degeneration, activating the caspase-mediated proteolytic system, exacerbates the atrophic phenotype.

Other factors whose expression and activity are mediated by innervation and are also associated with muscle denervation in ALS are neurite outgrowth inhibitor (Nogo)-A, Nogo-C and the nicotinic acetylcholine receptor (AChR) [19,20]. Nogo-A and Nogo-C expression, occurring early in ALS skeletal muscle, could cause repulsion and destabilization of the motor nerve terminals and subsequent degeneration of the axons and motor neurons. Real-time reverse transcriptase-polymerase chain reaction (RT-PCR) analysis (data not shown) did not reveal significant changes in Nogo-A and Nogo-C expression between wild-type and MLC/SOD1^G93A ^transgenic mice.

The AChR subunit mRNA accumulation and maintenance is also mediated by neural factors. It has been demonstrated that the γ-subunit mRNA level is tightly coupled to innervation. It is undetectable or low in innervated, normally active muscle and in innervated but disused muscle, whereas it is abundant along the whole fiber length in denervated muscle or in muscle in which the neuromuscular contact is intact but the release of transmitters is blocked [20]. Real-time PCR analysis for AChR-γ expression (Figure 2B) showed a slight but not significant increase in MLC/SOD1^G93A ^transgenic mice compared to wild type. Notably, the levels of AChR-γ expression were significantly and conspicuously upregulated in the SOD1^G93A ^muscle at paralysis stage (Figure 2B).

In addition, astrocyte activity, which is concomitant with motor neuron degeneration and normally increases at later stages of ALS disease [21,22], did not change between wild-type and MLC/SOD1^G93A ^spinal cord, while it selectively increased in the SOD1^G93A ^spinal cord at later stages of the disease as revealed by glial fibrillary acidic protein (GFAP) expression (Figure 2C). It seems likely that muscle atrophy is primarily associated with events related to the toxic effects of SOD1^G93A ^mutant protein, whereas motor neuron degeneration is a later event that contributes to exacerbation of the atrophic phenotype, promoting paralysis and muscle wasting.

### Analysis of molecular pathways involved in SOD1^G93A^-induced muscle atrophy

Transcriptional upregulation or downregulation of atrophy-related genes is a characteristic feature of muscle atrophy [23]. Several triggers and signaling proteins have been postulated as regulators of muscle atrophy, and it is often not clear whether muscle atrophy is the result of a reduction in protein synthesis, an increase in protein degradation or the combination of the two. Akt represents the critical signaling involved in protein synthesis and muscle hypertrophy [23]. Under different pathological conditions, Akt is dephosphorylated and its activity is reduced below control levels, leading to activation of atrophic pathways [24]. We analyzed the Akt expression, activity and cascade in both 16-week-old wild-type and MLC/SOD1^G93A ^mice.

**Figure 3:**
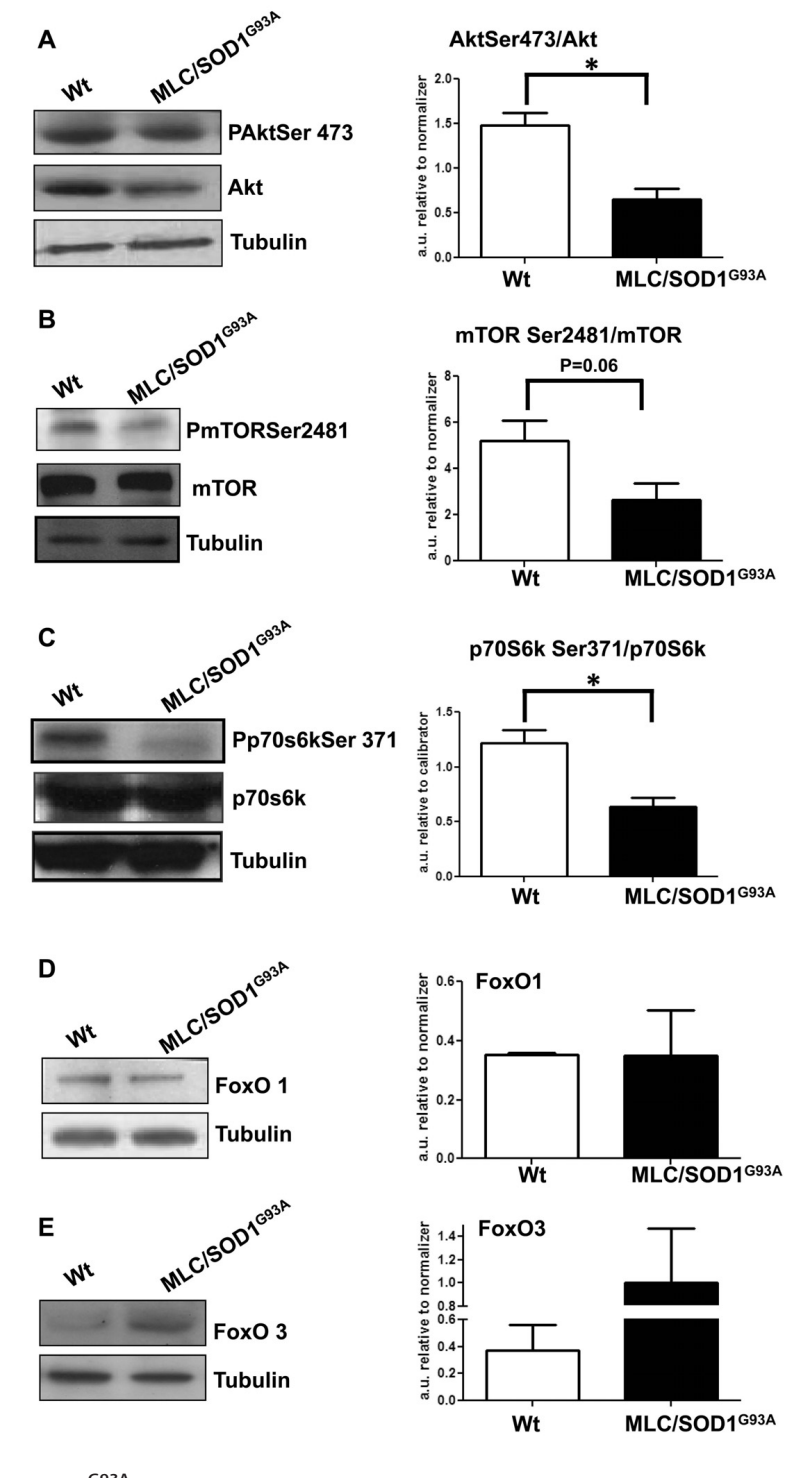
**Local expression of SOD1^G93A ^induces muscle atrophy via the negative modulation of the Akt pathway**. (**A-C**) Immunoblot analysis (n = 5) of total Akt and the phosphorylated form of Akt, mammalian target of rapamycin (mTOR) and p70S6K proteins in muscles of 4-month-old control (WT) and MLC/SOD1^G93A ^mice. (**A**) Akt phosphorylation (pAkt) was significantly reduced in MLC/SOD1^G93A ^muscle, with a slight change in total Akt levels compared to WT muscle. Right: Densitometric analysis of the ratio between total Akt and the phosphorylated form of Akt. (**B and C**) The phosphorylation levels of the downstream Akt intermediates, (**B**) mTOR and (**C**) p70S6K, resulted in significant downmodulation in the muscle of MLC/SOD1^G93A ^mice. Right: Densitometric analysis of five separate immunoblot experiments showing the ratio between total and phosphorylated forms of (**B**) mTOR and (**C**) p70S6K expression. (**D and E**) Representative Western blot analysis of (**D**) forkhead box O1 (FoxO1) (n = 5) and (**E**) FoxO3 (n = 3) expression in muscles of both WT and MLC/SOD1^G93A ^transgenic mice. Right: Densitometric analysis for FoxO1 and FoxO3 expression. Immunoblotting for α-tubulin served as a control for protein loading. *P < 0.05. Mean values ± SEM for pAkt/Akt: WT = 1.48 ± 0.14 and MLC/SOD1^G93A ^= 0.66 ± 0.12. Mean values ± SEM for pmTOR/mTOR: WT = 5.21 ± 0.88 and MLC/SOD1^G93A ^= 2.65 ± 0.73. Mean values ± SEM for p-p70/p70: WT = 1.21 ± 0.12 and MLC/SOD1^G93A ^= 0.64 ± 0.08. Mean values ± SEM for FoxO1: WT = 0.35 ± 0.01 and MLC/SOD1^G93A ^= 0.35 ± 0.15. Mean values ± SEM for FoxO3: WT = 0.37 ± 0.19 and MLC/SOD1^G93A ^= 1 ± 0.47.

Figure 3A shows that the absolute ratio of pAkt to Akt protein was significantly reduced in MLC/SOD1^G93A ^transgenic muscle compared with wild type. A downstream effector of the Akt pathway is the mTOR kinase, which in turn promotes the phosphorylation of p70S6K, leading to an increase in protein synthesis. Western blot analysis revealed significant downregulation of the phosphorylation levels of both mTOR and p70S6K intermediate in MLC/SOD1^G93A ^transgenic muscle, compared with that of wild-type littermates (Figures 3B and 3C).

In several atrophic conditions, the inhibition of the Akt pathway also leads to the activation of the FoxO members, which are able to promote muscle atrophy when overexpressed in cells and in whole muscle [24]. Western blot analysis (Figures 3D and 3E) revealed a selective accumulation of the dephosphorylated form of FoxO3, but not of FoxO1, in MLC/SOD1^G93A ^transgenic muscles compared to those of wild-type mice.

These data suggest that muscle atrophy induced by local SOD1^G93A ^expression is the result of the deregulation of the Akt pathway. This leads to two distinct responses: (1) an inhibitory effect on translation via the inhibition of mTOR and the dephosphorylation of p70S6K and (2) the activation of the FoxO3 pathway.

## Conclusions

The present work provides new insights concerning the control of muscle fiber size and the mechanisms of atrophy in animal models of ALS. Motor neuron degeneration and muscle atrophy are the major pathological processes associated with ALS, suggesting that nerve activity plays an important role in muscle homeostasis and remodeling. However, whether muscle atrophy associated with ALS is independent from motor neuron degeneration or results from it remains to be defined. We recently generated a novel transgenic mouse model in which the mutant SOD1^G93A ^gene involved in a familial form of ALS was selectively expressed in skeletal muscle [6]. We demonstrated that local expression of SOD1^G93A ^was sufficient to promote muscle atrophy and to impair the functional performance of skeletal muscle [6].

In the present study, we have extended previous work with the aim of defining the potential signal transduction pathways activated in response to SOD1^G93A^, expressed either locally [6] or ubiquitously [5], that cause muscle atrophy. In particular, in this study we have demonstrated that muscle atrophy in SOD1^G93A ^mice is an early event that is independent from motoneuron degeneration. The mechanisms responsible for the promotion of muscle atrophy involve a deregulation of Akt pathways and the activation of FoxO pathways, whereas caspases are selectively activated upon denervation or motoneuron degeneration.

**Figure 4:**
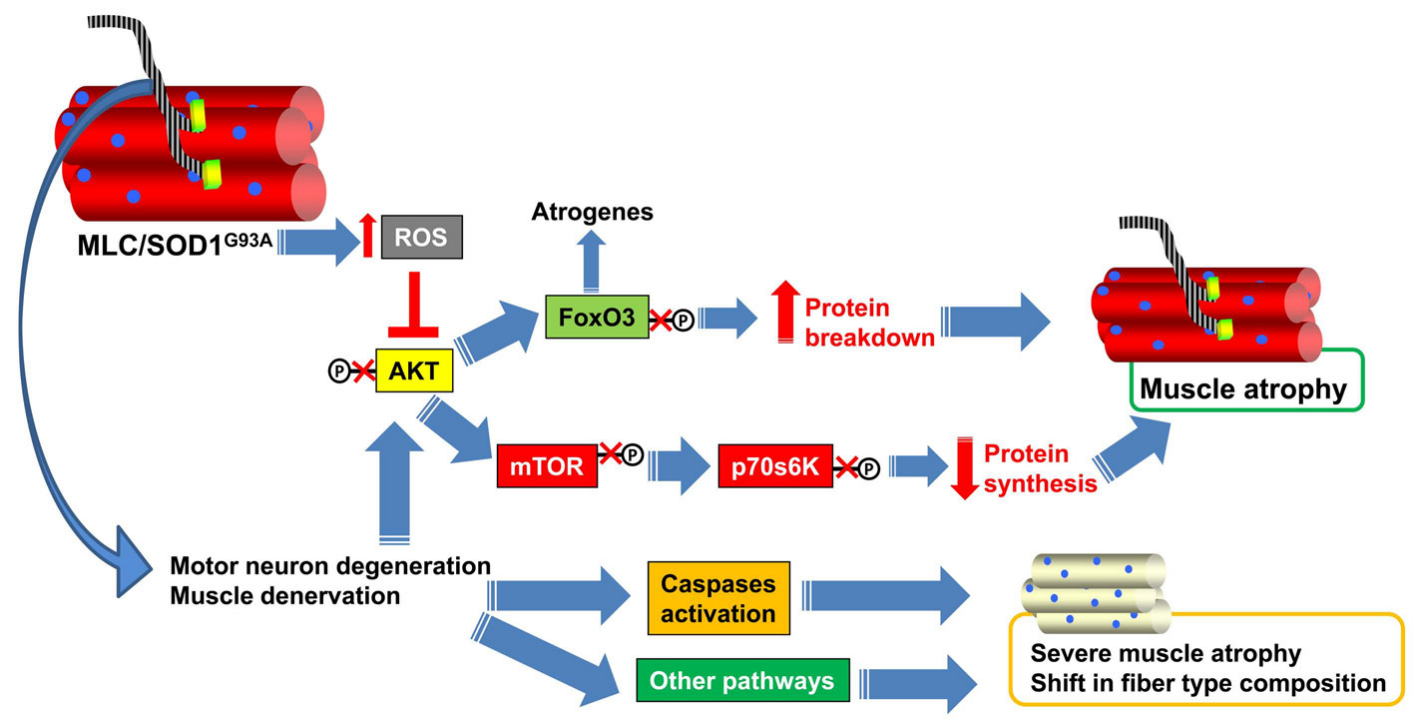
**A summary of the signal transduction pathways activated in SOD1^G93A ^transgenic mice**. The toxic effects of SOD1^G93A ^induce a local accumulation of reactive oxygen species which affects the Akt pathways, promoting muscle atrophy. The later motor neuron degeneration leads to more severe muscle atrophy and to a shift in fiber-type composition (see text of the results section for details).

The results in this study are consistent with a model (Figure 4) whereby muscle atrophy is an early pathological event that does not involve the expression and activity of caspases. In particular, the expression of SOD1^G93A ^promotes a reduction of the phosphatidylinositol 3-kinase (PI3K)/Akt pathway, leading to a reduction in protein synthesis and activation of the FoxO transcription factor. The two molecular branches of Akt activity might in tandem promote muscle atrophy at the early stage of ALS disease.

This initial morphofunctional alteration of skeletal muscle might be followed by NMJ destruction, distal axonopathy, astrocytosis in the spinal cord and finally motor neuron loss. This leads to a late exacerbation of muscle atrophy caused by caspase activation. Further work will define whether and how skeletal muscle affects the nervous system.

## Methods

### Mice

SOD1^G93A ^transgenic mice (Jackson Laboratory, Bar Harbor, ME, USA) express the human mutant SOD1^G93A ^allele containing the Gly93RAla (G93A) substitution, which is driven by its endogenous human promoter [5]. MLC/SOD1^G93A ^mice express the human mutant SOD1^G93A ^transgene under the transcriptional control of muscle-specific promoter (MLC), and its expression is selectively restricted in skeletal muscle. The animals were housed in a temperature-controlled (22°C) room with a 12:12 hour light-dark cycle. All procedures involving the use of animals were performed following experimental protocols approved by the Italian Ministry of Health (ISS) according to dlgs. 116/92 of European Economic Community Directive 609/86.

### Protein extraction and Western blot analysis

Protein extraction was performed in lysis buffer (50 mM Tris HCl, pH 7.4, 1% wt/vol Triton X-100, 0.25% sodium deoxycholate, 150 mM sodium chloride, 1 mM phenylmethylsulfonyl fluoride, 1 mg/ml aprotinin, 1 mg/ml leupeptin, 1 mg/ml pepstatin, 1 mM sodium orthovanadate and 1 mM sodium fluoride). Equal amounts of proteins (100 μg) from each muscle lysate were separated in sodium dodecyl sulfate polyacrylamide gel and transferred onto a Hybond-C Extra nitrocellulose membrane (GE Healthcare Life Sciences, Little Chalfont, Buckinghamshire, UK). After blocking with 5% nonfat dry milk, membranes were blotted with antibodies against caspase-3, Akt, pAkt, P70s6K, pP70s6K, FoxO1, FoxO3 (Cell Signaling Technology, Danvers, MA, USA), GFAP (Dako, Glostrup, Denmark) and tubulin (Sigma, St. Louis, MO, USA). The membranes were washed in Tris-buffered saline and Tween 20 (TBS-T) and incubated with horseradish peroxidase secondary antibody (diluted 1:10,000 in TBS-T) for 1 hour. Protein detection was carried out with SuperSignal Chemiluminescent Substrate (Pierce Biotechnology, Rockford, IL, USA).

### Caspase activity

Caspase activity was evaluated with the caspase-1 and caspase-3/ICE Colorimetric Protease Assay Kit (MBL International, Woburn, MA, USA). The assay is based on spectrophotometric detection of the chromophore *p*-nitroanilide (pNA) after cleavage from the labeled substrate YVAD-pNA. Protein extracts from wild-type, SOD1^G93A ^and MLC/SOD1^G93A ^mice were incubated with YVAD-pNA substrate at 37°C for 2 hours. The pNA emission was quantified with a spectrophotometer at 400 nm.

### Immunofluorescence analysis

For immunofluorescence analysis, 7-μm-thick muscle cryosections were fixed with 4% paraformaldehyde, washed in phosphate-buffered saline with 1% bovine serum albumin and 0.2% Triton X-100, preincubated for 1 hour in 10% goat serum at room temperature and incubated overnight at 4°C with primary antibodies MyHC-slow and laminin (Sigma).

### RNA extraction and quantitative reverse transcription polymerase chain reaction

Total RNA was prepared from liquid nitrogen powdered tissues homogenized in TRIzol reagent (Invitrogen, Carlsbad, CA, USA). Total RNA (1 μg) was treated with DNase I, Amplification Grade (Invitrogen, Carlsbad, CA, USA), and reverse-transcribed using the SuperScript™ III reverse transcriptase (Invitrogen, Carlsbad, CA, USA). Quantitative PCR was performed using ABI Prism 7000 Sequence Detection System software (Applied Biosystems, Carlsbad, CA, USA), and TaqMan^® ^Universal Master Mix (Applied Biosystems, Carlsbad, CA, USA) for real-time RT-PCR amplification. Quantitative RT-PCR sample values were normalized for the expression of β-actin and reported as arbitrary units.

### Statistical analysis

The Mann-Whitney *U *test was used for comparison among the different experimental models.

## Abbreviations

AChR: nicotinic acetylcholine receptor; ALS: amyotrophic lateral sclerosis; GFAP: glial fibrillary acidic protein; MyHC: myosin heavy chain; NMJ: neuromuscular junction; SOD1: superoxide dismutase 1; WT: wild type.

## Competing interests

The authors declare that they have no competing interests.

## Authors' contributions

AM designed experiments. GD and MA carried out the experiments and analyzed the data. AM wrote the paper. All the authors read and approved the final manuscript.
